# Partnerships in nursing homes: How do family caregivers of residents with dementia perceive collaboration with staff?

**DOI:** 10.1177/1471301220962235

**Published:** 2020-09-25

**Authors:** Linda JM Hoek, Jolanda CM van Haastregt, Erica de Vries, Ramona Backhaus, Jan PH Hamers, Hilde Verbeek

**Affiliations:** Department of Health Services Research, Care and Public Health Research Institute (CAPHRI), 5211 Maastricht University, Maastricht, the Netherlands

**Keywords:** partnership, communication, family caregiving, nursing home, dementia

## Abstract

**Background:**

Partnerships between family and nursing staff in nursing homes are essential to address residents’ needs and wishes. Collaboration is needed to create partnerships; nonetheless, challenges exist.

**Aim:**

This study aimed to gain insights into the experiences of families collaborating with staff.

**Method:**

Semi-structured interviews were held with 30 family caregivers of nursing home residents with dementia.

**Findings:**

Data reflected three themes, which shaped collaboration with staff from families’ perspective, ‘communication’, ‘trust and dependency’ and ‘involvement’.

**Discussion:**

Good communication appeared to be a requisite condition for having trust in staff and quality of involvement in residents’ life. Good communication was described as having informal contact with staff, which enabled family and staff to build a personal connection. Consequently, this seemed to increase trust and satisfaction regarding involvement.

**Conclusion:**

Findings suggest that increasing informal contact and building a personal connection should be a priority for staff in order to improve collaboration and to create partnerships with families.

## Introduction

When dementia progresses, people become increasingly reliant on their social environment to meet their needs and wishes (Smaling et al., 2018). When care at home becomes too complex, people with dementia may move to a nursing home, where they receive more intensive, specialised care among other residents with dementia (Sanford et al., 2015). Nonetheless, family involvement and continuation of family roles remain essential for residents to be able to live the life they want, as families and their relatives have developed a relationship over time (McCormack & McCance, 2006; Puurveen et al., 2018). However, families often experience uncertainties regarding their role after the person with dementia moved in to a nursing home, as it often contains more than solely visiting (Baumbusch & Phinney, 2014). After moving into a nursing home, families take on other roles and are less involved in physical care and decision-making (Baumbusch & Phinney, 2014). As advocates, family caregivers can work together with nursing home staff by sharing information on the resident’s biography, meaningful activities, and preferences in care and daily life (Powell et al., 2018). In addition, they can also be involved in making decisions regarding care plans and provide emotional and instrumental support (Ryan & McKenna, 2015).

It is challenging in nursing homes to build a partnership between family and nursing staff, in which both collaborate as partners in care (Holmgren et al., 2013; Legault & Ducharme, 2009; Ryan & McKenna, 2015). Nursing home staff often find it difficult to collaborate with family caregivers and have been known to consider the family to be ‘difficult’ and demanding (Bauer et al., 2014; Givens et al., 2012). In addition, nursing staff often perceive the nursing home as their working arena (Holmgren et al., 2013). Therefore, collaboration and communication with family caregivers might be perceived by nursing home staff as having to justify work tasks. Another aspect that interferes with collaboration is that, in general, nursing home staff feel that family caregivers may not take into account that they provide care to a group of residents living together, instead of solely caring for individuals (Bauer, 2006). On the other hand, challenges exist from the perspective of family caregivers because it might be difficult to collaborate with staff as equal partners. For example family caregivers are commonly seen only as visitors by the nursing home staff and are not always involved in resident care plan changes (Holmgren et al., 2013; Reid & Chappell, 2017). In addition, because nursing staff regularly work according to care and administrative tasks and routines, it might be difficult for family caregivers to initiate contact with nursing home staff because this may require interrupting staff routines (Bauer & Nay, 2003; Wilson et al., 2009). Furthermore, nursing staff usually take over physical care tasks after residents’ admission to a nursing home (Gaugler et al, 2010; Smaling et al., 2018). Hence, family caregivers might expect that staff will provide all the care and that this care is of high quality (Kiljunen et al., 2018). Nonetheless, to be able to address residents’ needs and wishes, and for residents to maintain and engage in social relationships, it is important to provide care from a relationship-centred perspective with positive relationships between residents, family caregivers and staff (McCormack et al., 2012).

There is a need to facilitate a partnership between family and staff to fulfill residents’ needs and wishes within current practice. These partnerships need to underline the importance of reciprocity between family and staff, by creating mutual respect and acknowledging each other’s important contribution, as equals in care. Nonetheless, challenges exist and evidence on intervention programs that encourage or create partnership between family caregivers and nursing home staff is scarce. Recent studies on improving family involvement within nursing home care mainly target interventions that address specific goals, such as decision-making at the end of life stage and advanced care planning (Agar et al., 2017; Smaling et al., 2018). Only three broader intervention programs aimed to improve collaboration and partnership between family members and nursing staff in nursing homes (Maas et al., 2004; Robison et al., 2007; Zimmerman et al., 2013). Two intervention programs targeted family involvement within dementia-specific facilities. These were designed to involve families within nursing home care by aiming at negotiating partnership (Maas et al., 2004) and to improve collaboration and communication between family and staff (Robison et al., 2007). One intervention aimed at helping family caregivers to identify activities in which they could participate and to help them to create a role for themselves (Zimmerman et al., 2013). These three intervention programs addressed the importance of finding ways to involve family within nursing homes. Nonetheless, these did not emphasise creating partnerships between family and staff to jointly support residents in living the life they want, which needs to be a central starting point within providing relationship-centred nursing home care. As nursing staff members frequently report difficulties in collaborating with family caregivers (Bauer et al., 2014), more knowledge is needed on how family experiences partnerships in order to improve interventions that aim at building a partnership between family and staff that recognises both equal important contributions to resident’s life. Up to now, far too little attention has been paid to family caregiver’s perspective. Exploring their perspective on current partnership needs to be established before interventions that aim to build a partnership between family and staff can be improved.

In recent years, radical alterations have been made to create a homelike environment, including family members within the part of the caregiving process, in order to better address residents’ needs and wishes (Doetter & Schmid, 2018; Gräske et al., 2015; Zimmerman et al., 2016). These initiatives have characteristics that may improve family members’ involvement with nursing staff, such as a small-scale homelike environment, more opportunities to interact with staff because of smaller caseload levels and more resident autonomy. For example previous research showed that family members had more personal contact with staff and were more intensively involved in everyday household activities in small-scale nursing homes (Verbeek et al., 2012). Furthermore, nursing staff working in small-scale, homelike care environments showed better listening skills towards residents compared with those in more institutional, large-scale nursing home wards (de Rooij et al., 2012).

Since alterations have been made within nursing home care, as part of the culture change movement, more knowledge on how family caregivers currently experience collaboration and partnership within the care for their relative with staff is needed. This study aimed to provide new insights into experiences of family caregivers of nursing home residents with dementia with collaboration between them and the nursing staff, and how their experiences and preferred roles are being involved. This knowledge could support care organisations worldwide with improving collaboration and with developing solid partnerships between family caregivers and nursing staff in the nursing home. This study will contribute to a deeper understanding of factors that are important when developing new interventions aimed at involving family caregivers of nursing home residents with dementia and developing partnerships between family and staff.

## Method

### Design

For the purpose of this study, a qualitative descriptive research design was chosen. Data were collected through semi-structured interviews with family caregivers of nursing home residents with dementia. The COREQ guideline was used in reporting information on this study (Tong et al., 2007).

### Participants

Family caregivers of nursing home residents were recruited to participate in the study. The eligibility criteria for family caregivers were providing care for their relative with dementia who lived in a nursing home. In the Netherlands, a standardised assessment procedure is carried out by a governmental agency, and in accordance with the residents’ family or legal guardian, before a person is admitted to a nursing home. The sampling procedure focussed on including a variety of perspectives from different nursing home environments. Therefore, family caregivers were recruited from five different nursing home wards, within three different psychogeriatric care organisations, in the south of the Netherlands, based on convenience sampling. We aimed a sample of about five family caregivers per ward, that is 25 interviews in total. Two large-scale wards and three small-scale wards were included in this study. Within these large-scale wards, care was provided for 18 to 21 residents, and daily life was mostly determined by routines in reference to the institution and what fit the group of residents and/or staff; small-scale wards aimed to provide care within a homelike, personal environment for six residents, where activities were integrated into daily life. In four study sites, regular changes in staff members were existent. All 58 main contact persons, for example family caregivers who function as contact between resident’s family and the ward, for residents with dementia within the chosen wards were invited to participate in the study. These main contact persons were chosen, as of all family caregivers, they are often the most present at the ward and have the first and most contact with staff. They were given the opportunity to either participate themselves or to invite another family caregiver (along) to participate.

Of the 58 family caregivers invited to participate in the study, 30 agreed to be interviewed, 11 refused to participate and 17 family caregivers did not return the consent form. Table 1 provides an overview of family caregiver characteristics. Nineteen family caregivers from two large-scale wards participated in the study, and 11 family caregivers from three small-scale wards participated. Although data saturation was not the guiding principle in the sampling process, after about 20 interviews, redundancy in the data was identified.

**Table 1.** Family caregiver (*N* = 30) characteristics.

	Family caregiver
Characteristic	*M* (*SD*)
Age (years)	57.12 (5.41)
	*n* (%)
Gender	
Female	27 (90)
Male	3 (10)
Relationship with relative	
Daughter	24 (80)
Son	3 (10)
Spouse	1 (0.03)
Niece	1 (0.03)
Legal representative	1 (0.03)
Visits relative more than once a week	27 (90)
Nursing home facility	
Large-scale ward	19 (63)
Small-scale ward	11 (37)

### Procedure

Prior to data collection, the researcher informed family caregivers about the goal and content of the study during an information meeting which was facilitated by the ward manager. Written information on the study and an informed consent form were handed out subsequently. Family caregivers who were not able to attend the information meeting received written information on the study and an informed consent form by mail, which was sent by the ward manager. On obtaining written informed consent from the family caregivers, the researchers called them to plan an interview. Interviews were held between February and October 2017. The interviews lasted 20–50 minutes, with one exception of an interview that lasted about 120 minutes. In an attempt to make each interviewee feel as comfortable as possible, a second relative of the resident was allowed to participate in the interview, as three interviewees preferred this. Also, the interview took place at a location that was convenient for the family caregiver: for example at their home, the nursing home or at Maastricht University. Before interviewing the family caregivers, the purpose of the research was clearly explained and family caregivers were assured that the data would be treated confidentially.

### Data Collection

Semi-structured interviews were conducted to collect data on the experiences of family caregivers of nursing home residents with dementia with regard to their collaboration with nursing home staff. Background information of the family caregivers was collected – specifically, gender, age, relationship to the resident (spouse, child or other), frequency of visitations and the amount of months the resident had been at the nursing home. Three researchers performed the interviews (LH, EdV and RB) using a standardised interview guide. The interview started with a comprehensive question with regard to feeling at home to gather a first sense of the family caregiver’s opinions about the ward in general. Consequently, more specific questions on their experiences with collaboration – including the things that hinder or facilitate it – were asked. Interview questions are presented in Table 2. Probing questions such as ‘Can you elaborate more on (…)?’ or ‘Why do think so?’ were asked to obtain more in-depth information. The interview data were recorded on a digital audio recorder and transcribed verbatim.

**Table 2.** Interview questions.

Topic (theme)	Question (example)
Comprehensive/introductory questions	Would you say you feel at home or welcome at the ward? (Do you feel at ease?)
	What makes you feel at home or welcome? What contributes to or hinders this?
Specific questions regarding collaboration with nursing home staff	How would you describe the collaboration between you and nursing home staff at the facility where your loved one lives?
	Which things do you work together on?
	Are you able to do everything you would like to do for your relative?
	Are you being invited by the nursing home staff to participate in care or other activities?
	What aspects regarding collaboration with nursing home staff are going well?
	What aspects regarding collaboration with nursing home staff could be improved upon, according to you?

### Data analysis

The analysis contained a qualitative thematic approach (Moser & Korstjens, 2017). Two researchers (LH and EdV) and two research assistants compiled the audio data by transcribing the interviews; this process achieved closeness and familiarity with the data. Subsequently, transcripts were coded so as to convert raw data into meaningful groups of text. This included identifying ideas, concepts and themes that were connected to one another and giving them a definition or code. A codebook was developed, containing all codes that related to the purpose of the study. Qualitative data analysis software MAXQDA was used in assisting the researchers to structure the process and to visually organise the coding process. After all information from the transcripts was broken down into codes, the researchers sought to find (sub)themes across all codes. All themes were then critically checked for content and quality in order to ensure consistent relationships. Thereafter, interpretations were made across themes, and patterns within the data were identified. Finally, conclusions were drawn with regard to answering the research question.

### Rigour

This study established rigour by including a variety of nursing home wards in order to obtain different views on the topic. To increase the reliability of the analysis, the whole team was involved during the data analysis process as a form of analyst triangulation (Guion et al., 2002), meaning that perspectives from the six different researchers were compared. For example interviews were conducted by three researchers (LH, EdV and RB). Next, audiotaped data were transcribed verbatim by two researchers (LH and EdV) and two research assistants. Four transcripts were read and coded by LH and discussed in detail with HV. Subsequently, all transcripts were coded according to an initial codebook by either LH or EdV and checked by the other in order to find (dis)agreements on interpretations in text fragments. Disagreements were discussed and an interpretation that best matched the meaning of what was expressed was agreed upon. Moreover, researchers LH, EdV and HV discussed all interpretations on a weekly basis. General interpretations of the data were discussed within the whole team. The research team consisted of six researchers who all had experience in research in the field of dementia and long-term care. However, they had different fields of expertise: three in (clinical) psychology, one in nursing, one in health policy and one in quality improvement. This promoted a deep understanding of the context and interpretation of the phenomenon in current nursing home practice.

### Ethical considerations

Ethical approval for this study was obtained from the Ethics Committee of Zuyderland-Zuyd (No. 16-N-233). Family caregivers voluntarily signed informed consent after they were fully informed about the purpose and procedures of the study. They were reassured that data would be kept confidential and that they could discontinue their participation at any time during the study.

### Findings

The data reflect three themes that shaped collaboration with nursing home staff from the perspective of family caregivers (Table 3). The theme, ‘communication’, appeared to be a requisite condition for creating trust and quality of involvement, which were part of the themes ‘trust and dependency’ and ‘involvement’.

**Table 3.** Collaboration: themes, content and description.

Theme	Content	Description
Communication	Informal contact, need for contact, informing and inviting	A way of having contact or interaction with staff, either formal or informal
Trust and dependency	Monitoring and personal connection	Feeling highly responsible for a relative while being dependent on staff care provision and deciding whether to trust staff by monitoring and creating a personal connection
Involvement	Advocacy, visiting, and caring activities	Being involved in relative’s daily life by means of looking after the relative and keeping control over relative’s daily life by visiting and caring for the relative

Most family caregivers expressed that they did not identify themselves as being part of a collaboration with nursing home staff. They described that they themselves performed individual tasks regarding care, which were separate from those performed by the nursing home staff. Almost all family caregivers assigned physical care tasks to nursing home staff, whereas social tasks, such as visiting and undertaking individual activities, they assigned to themselves. The themes are described in more detail below.

### Communication

Communication was of major importance for family caregivers when describing how they experienced collaboration. Communication was seen as a way of having contact or interaction with staff, which was considered a requisite condition for collaboration. Family caregivers described contact as being both formal and informal. Formal contact consisted of types of information and invitations, whereas informal contact was expressed in terms of having personal conversations.

#### Informal contact

A factor that the family caregivers considered as facilitating communication was having informal and personal dialogue with staff – for example being greeted when entering the room or staff asking, ‘How are you?’ These conversations, although small, were important to family caregivers. It made them feel welcome when visiting and made it easier for them to talk to staff. Family caregivers who felt welcome and at home described more positive experiences regarding communication with staff.

Because I know the staff and they know me. They also immediately see when something is wrong because I might look different. They ask ‘Is something wrong?’ It’s becoming a little family, you know. (Daughter (1), large-scale facility 1)

#### Need for contact

More than half of the family caregivers expressed a need for having more contact with nursing home staff than they currently had. However, some experienced barriers to communication with staff that prevented them from having the desired contact. Reasons for this varied. Some were afraid of being seen as ‘moaners’ and, therefore, kept their concerns to themselves because they said that they would not like to negatively comment on the care provided. In addition, some felt afraid of the consequences for their relative if they expressed criticism.

These are organization guidelines. It’s not up to me to interfere with them. I would not do that. It is in the best interest of my mother, so I have to watch what I’m saying and how I express things. I would not want them to think ‘Oh, it’s her again.’ That could be at the expense of their approach toward my Mom. I have to protect her a little. (Daughter (1), large-scale facility 2)

Moreover, some argued that they would not seek interaction with staff because they would not want to be a burden on staff by interrupting their routines. In addition to these barriers regarding beliefs, low visible staff presence and having unfamiliar staff taking care of their relatives hindered communication, according to family caregivers. At some wards, family caregivers mentioned a high staff turnover, which made it difficult for family to find ways to engage in communication with new staff. This was mostly evident in one ward where staff would retreat into a private office with a closed door.

The staff are busy doing their own things and they have loads of work to do. They don’t have time to talk with people who enter the ward. The reception is completely deserted; only occasionally does someone sit there. There is no sense of making contact with people who come in. (Daughter (2), large-scale facility 2)

In addition, some family caregivers described that it was not always possible to contact staff because they faced difficulties contacting staff outside of secretary office hours.

#### Informing and inviting

Family caregivers mentioned two forms of communication between family caregivers and nursing home staff, that is informing and inviting. Most family caregivers described examples of how they informed nursing home staff and vice versa, either at the staff’s request or spontaneously. For example family caregivers informed nursing home staff about how their relatives like to be cared for, their relatives’ health concerns, having clothing items in need of laundering, or when they wanted to show appreciation towards staff.

I complimented it. I came over last week and said ‘May I ask who provided care for her this morning?’ I said, ‘Look how nice Mom looks right now. It’s so nice to see her looking so well.’ (Daughter (2), large-scale facility 1)

Correspondingly, all family caregivers described that staff often informed them either about medical issues, such as treatment updates, visitations from the elderly care physician, and falls, or about practical issues, such as running out of soap or torn leggings. Family caregivers mentioned that staff provided this information often via telephone or email, depending on the seriousness. A few family caregivers expressed that staff would occasionally inform them about how their relative spent his or her day or whether or not their relative enjoyed the day or showed signs of agitation.

In addition to communicating through information, most family caregivers also described communication through invitation. They expressed how staff invited them to participate in organised communal activities, such as going to the zoo, musical evenings, or Christmas dinner. Family caregivers expressed that staff mostly displayed invitations on the ward’s noticeboard, through a public letter, or generic email. Family caregivers from one ward said that the manager would also personally invite family caregivers, which they appreciated. By contrast, family caregivers from another ward mentioned that they were not invited to participate in any activities.

They were interns, I guess, who I didn’t know. If they had asked family caregivers, someone would have signed up to participate. If they had asked me, I would have joined and I think others would have too. (Daughter (3), large-scale facility 1)

A few family caregivers expressed that they recalled being invited to talk about their relative’s wishes and needs during the intake at admission, but rarely afterwards. Few family caregivers mentioned having occasional informal contact, such as personal contact that does not regard care and entails contact at a personal level. These few family caregivers and others who often communicated with staff expressed positive beliefs regarding care and were more satisfied with the ward in general, as opposed to other family caregivers.

### Trust and dependency

Having trust was one of the important aspects to good collaboration. Family caregivers reflected on issues that were related to having trust in nursing home staff and how this was experienced, while being dependent on staff with regard to care provision for their relative. Family caregivers perceived that they were dependent on staff, as staff mostly determined the resident’s daily life. Family caregivers felt highly responsible for their relative and, therefore, having trust in staff and how they take care of their relative was essential for them to establish a partnership.

#### Monitoring

A recurrent theme in the interviews was a sense amongst family caregivers that they had to monitor the resident care provided by staff. Family caregivers expressed a lack of trust regarding how care was provided, staff’s failure to honour agreements, and staff’s lack of communication with family regarding agreements and the status of these agreements.

For example she went to the dentist today to have her teeth cleaned. They (the staff) have to make efforts to clean them better, which we talked about earlier, after the last time she went to the dentist. You have to keep an eye on those things. (Daughter (1), small-scale facility 2)

Family caregivers who expressed that staff honoured commitments had more trust in staff. Subsequently, they were more satisfied with collaboration and had less concerns when thinking about their relative living in a nursing home.

#### Personal connection

Moreover, the majority of the family caregivers stated that it was important to them to have a personal connection with nursing home staff members. For example most family caregivers explained situations in which they would wait to ask certain things because the principal staff contact person was not present at the ward during their visit. Family caregivers reported that they would not ask other staff because they either did not know them, they knew other staff did not have the personal information, or they did not establish a relationship of trust with other staff and were not sure whether or not they could make agreements with them.

I notice that when I pass something on, it isn’t written down or it will be forgotten. Then, I’d prefer to go straight to our principal contact person because she writes it down and then everybody can read it. (Daughter (1), small-scale facility 1)

In addition, some family caregivers described feeling surprised when staff were receptive to receiving feedback because they did not expect this. Family caregivers described that when staff were open to receiving feedback, this was helpful for them to address issues and have small conversations in the future. This would make communicating easier because they experienced fewer barriers to addressing issues.

Furthermore, most family caregivers elaborated on how they passed on information to other family caregivers of their relative. Some also pre-discussed strategies with other family caregivers on how to negotiate issues with staff.

### Involvement

In describing collaboration with nursing home staff, family reflected upon own involvement in resident’s daily life by means of advocacy, visiting their relative and caring for their relative. Overall, the family caregivers demonstrated that, in general, they were satisfied with the quantity of their current involvement (e.g. frequency of visits and participation in resident’s daily life). However, the quality of their involvement (e.g. satisfaction with how they were involved by staff in resident’s daily life) could be improved upon.

#### Advocacy

Family caregivers described various ways in which they looked after residents’ interests as advocates. All family caregivers stated that everything they did was in the best interests of their relatives and expressed this, for example by saying, ‘My mother would have loved this or my mother would not have wanted this.’ Family caregivers did not literally mention that they would like to manage the resident’s life; however, they described their experiences with monitoring the resident’s life within the nursing home. For example they explained how they felt about certain situations and how they intervened, often out of dissatisfaction, or contributed when needed.

For example at a certain point, she broke her leg and took painkillers four times a day. At some point, I said, ‘Wouldn’t you decrease the amount of painkillers?’ They took my advice and discussed it with the doctor. It slowly decreased. I don’t know everything, but I thought, ‘Do you guys think about that?’ (Daughter (2), small-scale facility 1)

Most family caregivers mentioned how they contributed to residents’ life by performing practical tasks, such as buying clothes, washing clothes and decorating the resident’s room. Moreover, most family caregivers expressed the need to have more knowledge about the resident’s daily life. Nonetheless, they experienced little involvement with staff regarding care planning or discussing care plans and were not fully able to carry out their role as advocates in the care of their relative.

If we had a shared folder, we could leave comments on it and the communication could be shaped in that way, even if the principal contact person is around. I don’t care who I talk to; I just want to know that things are being done and that I can follow the decision process and what will be happening. (Daughter (4), large-scale facility 1)

#### Visiting

Almost all family caregivers perceived no restrictions about visiting. For example they could visit when wanted, they could walk or undertake trips outside of the ward with their relative when they wished and grandchildren were welcome anytime. A small number of those interviewed suggested that they were not able to do whatever they wanted because of unfamiliarity with some staff members due to staff changes. This made it difficult to know what they were allowed to do during visitation – for example did they have a right to spontaneously get coffee or fruit at the ward. In addition, a few interviewees, from both small-scale and large-scale wards, argued that they sometimes had the feeling that they were interfering with staff routines and ward rules when asking for something.

Sometimes my sister will take a look. I won’t call about difficult questions because there’s little chance that the person who picks up the phone has any idea what I’m talking about and she could have used that time to brush someone’s teeth or something. (Daughter (5), large-scale facility 1)

#### Caring activities

Family caregivers revealed small examples of collaboration regarding resident care. Most family caregivers would argue that caring tasks are the staff’s responsibility.

Often, when I go to my Mom, she is still in bed and I help her get up. Then, a member of staff walks in and says ‘You’re on a roll!’ I respond, ‘You can take over now!’ I will focus on other things but, to me, that is the staff’s responsibility, but there you go. (Daughter (3), large-scale facility 2)

In addition, they mentioned that tasks such as showering, helping residents to go to the bathroom and cutting nails were staff tasks. Only a minority of family caregivers mentioned that they performed care tasks when visiting. For example they described how they would help residents who were unable to eat independently with eating and drinking, help their relative to go to the bathroom or escort them to the hairdresser. Additionally, there were some family caregivers involved in tasks such as cooking or playing the piano at the ward, and some described that they were able to have dinner together with their relative. Except for one family caregiver, none of the family caregivers mentioned that they would organise activities for residents on their own initiative.

Furthermore, one family caregiver expressed that the manager introduced compulsory tasks for family caregivers, such as cooking or participating in activities. This, however, caused family caregivers to resist participation and become upset.

Even though family caregivers wanted to be involved in residents’ lives, only a few family caregivers stated that they experienced shared decision-making regarding preferences in daily routines, meaningful activities and medical decisions. Family caregivers who communicated often with staff were more satisfied about the decision-making process. They expressed that staff called them to inform them about medical or behavioural changes. In addition, some expressed that they were involved in care by being asked by staff to discuss how a certain situation regarding behaviour or care could best be resolved.

They called me and said, ‘We called the doctor. Can you come over? Maybe you can talk some sense into her because it’s getting dangerous.’ So, I went and talked to my Mom and participated in her delusion by justifying her thoughts and actions. Then, my Mom calmed down and went with me, and the staff member gave me a thumbs up and complimented me on how I handled the situation. (Daughter (2), small-scale facility 2)

## Discussion

This study has identified that family caregivers perceive collaboration by means of ‘communication’, ‘trust and dependency’ and ‘involvement’. Data suggest that communication is a requisite condition for having trust and affects the perceived quality of family caregivers’ involvement in the daily lives of their relatives. Family caregivers considered having informal contact with staff as good communication, which enabled family caregivers and nursing home staff to build a personal connection. Accordingly, this seemed to increase trust and satisfaction regarding quality of family caregivers’ involvement in the daily life of the resident.

Within all aspects of communications, the present study especially highlights the importance of having informal communication and creating a personal connection, in order for family caregivers to feel involved within the nursing home. Findings of our study indicate that these are requisite conditions for establishing trust and good quality of family caregivers’ involvement. In addition, the findings provide a deeper understanding of building a positive collaboration in nursing homes by enhancing good communication and increasing personal contact and trust between family and staff. Our findings are in line with a more general definition of collaboration, which can be defined as a dynamic, voluntary form of interaction or activity between two or more people (Morris & Miller-Stevens, 2016). It implies having interaction regarding goal setting and decision-making, within an environment of mutual trust, harmony and respect (D’Amouret al., 2005). Also, it was reported that sharing, partnership, interdependency and power are related concepts for collaboration in general (D’Amour et al., 2005; Morris & Miller-Stevens, 2016). In addition, open and honest communication, being attentive to each other, and valuing each other’s perspective and contributions appear to be important elements regarding developing partnerships between people (D’Amour et al., 2005; Stichler, 1995). Especially in nursing homes, collaboration and partnership between family and staff are needed in order to address residents’ needs and wishes. Hence, because family caregivers within this study expressed a need for contact, there is a need to develop an intervention that is targeted at combining both family caregivers and nursing staff. In that way, family and staff can optimally share personal and professional knowledge to improve residents’ daily lives.

Our study provides deeper insights into the importance of having a fixed nursing home staff team, for example having low staff turnover, which is essential for family caregivers to collaborate with staff and build a partnership. Family caregivers within the current study experienced difficulties building a collaborative environment with new staff members. As family caregivers felt responsible for their relative and were dependent on nursing staff regarding care for their relative, the sometimes complicated process of building trust restarted with the introduction of new staff. These findings add to previous research which underlines the complexity of having to trust others with care (Shanley et al., 2011) and building a partnership because it is suggested that staff might perceive family caregivers as difficult and describe interactions as complicated and time-consuming (Bauer et al., 2014; Holmgren, et al., 2013; Utley-Smith et al., 2009). This might further complicate building partnerships. Subsequently, collaborating by means of ‘us’ and ‘them’ is problematic when developing partnerships (Holmgren et al., 2013) and might result in low levels of communication, while family caregivers within this study expressed a need for having more contact. What is more, building a partnership is most likely a continuous, challenging and ongoing process, starting from the phase of the resident moving into the nursing home to facing challenges in collaboration as dementia progresses. Especially then, good communication is imperative.

Furthermore, our study’s findings show that family caregivers perceive their role within the nursing home as much more than being ‘merely a visitor’. However, they do not regard themselves as true partners in care. This is in line with previous research from Baumbusch and Phinney (2014), who also found that family perceives that their role within the care for their loved one is much broader than only visiting. Moreover, the findings of this study have extended our knowledge of how family involvement is perceived by exploring the perspectives of family caregivers. This was important because existing interventions regarding improving family involvement were mainly developed based on knowledge from staff’ perspective. In order for family caregivers to be partners in care instead of visitors, findings from this study suggest that family caregivers need to be involved as equals in decision-making to establish a partnership. Within all aspects of resident’s daily life, family’s important role and involvement should be acknowledged as equally important as staff’ role, as staff’ role currently holds more power. Especially since earlier research on staff’ perception of family involvement in nursing homes shows that, even though staff consider the importance of incorporating the role of family within daily care (O’Shea et al., 2014), they often perceive family caregivers as non-experts with regard to medical care and decision-making (Holmgren et al., 2013). Besides, due to high staff workloads and time pressure (Haesler et al., 2010; Majerovitz et al., 2009), communicating with family caregivers, and especially with those who are perceived as ‘difficult’ and ‘demanding’, is most likely not regarded as a priority by nursing home staff. Additionally, family caregivers within our study expressed that they still perceive the nursing home as the staff’s territory and described being dependent on staff in various ways with regard to care for their relative. However, family caregivers did not express that they were aware of staff’s perspective on the importance of family involvement, while the staff is dependent on family in various ways in order to provide the best care.

### Limitations

Some limitations regarding this study should be considered. While a variety of nursing home wards were included in the study in order to enhance result’s contextuality and increase transferability, we included a considerable number of daughters. It is unfortunate that this study did not include more views of spouses of residents, who might collaborate with staff in different ways because they have a different relationship with the resident (Pot et al., 2001). Notwithstanding the relatively limited number of included spouses or sons, this work offers valuable insights into current practice because women are predominantly represented within care as family caregivers (Lagault & Ducharme, 2009; Holmgren et al., 2013). Furthermore, as including views from a variety of nursing home wards was aimed for, data saturation was not a guiding principle in the sampling process. For the purpose of this study, therefore, we might have interviewed less participants as data saturation was decided upon after about 20 interviews. Finally, this study did not incorporate the views of nursing staff; including the views of staff could have contributed to providing recommendations for collaboration in practice, based on the comparison of both views.

### Implications for further research

The findings of this study have implications for further research. First, further research is needed to develop and evaluate intervention programs that create a partnership between family and staff, regarding the nursing home care for residents with dementia. It needs to be identified which staff competencies are needed and how nursing home staff can collaborate with people holding other interests, in order to establish communication, trust and both frequency and quality of family involvement. Interventions need to focus on building a partnership around residents’ needs and whishes, by improving informal and formal communication and developing a personal connection between family caregivers and nursing staff. In addition, these need to emphasise reciprocity, by targeting both family caregivers and staff and acknowledging each other’s important contribution and being equal partners in care. Furthermore, as nursing staff’s positive attitude towards developing partnerships is crucial, further research needs to focus on whether the nursing home environment is able to provide a trusting and collaborative atmosphere.

### Implications for practice

Our findings indicate that, in particular, having informal communication is important as this is a way to build a personal connection and trust between family caregivers and nursing home staff. Based on this study, it is suggested that nursing staff should be aware of the positive effects of welcoming family, approaching them personally, being friendly and look for ways to intensify the quantity and quality of informal contact, in order to build a personal connection. Additionally, good communication requires going beyond sharing information on medical status and daily care. Instead, combining improving formal and informal ways to communicate must be considered. Digital platforms such as social media might enhance contact and improve relationships between family and staff (Caughlin et al., 2016), for example by sharing experiences with residents’ daily life within the nursing home. Consequently, family caregivers need to be given the opportunity to be engaged within resident daily life by nursing home staff. In addition, they need to be supported within decision-making regarding medical decisions and discussions (Carter et al., 2018) to become a full and equal member of the team. Therefore, it is of major importance for staff and family caregivers to engage in dialogue and find new ways to communicate and collaborate to improve residents’ daily lives.

## Conclusion

Findings of our study suggest that in the opinion of family caregivers, staff mostly determine residents’ daily life. Staff might not always be aware of the importance of family involvement. Furthermore, family caregivers might not consider their role as equal partners in care. Therefore, increasing informal contact and building a personal connection should be a priority for staff in order to improve collaboration and to create partnerships with families.
